# *Candida albicans* Pathogenicity and Epithelial Immunity

**DOI:** 10.1371/journal.ppat.1004257

**Published:** 2014-08-14

**Authors:** Julian R. Naglik, Jonathan P. Richardson, David L. Moyes

**Affiliations:** Mucosal and Salivary Biology Division, King's College London Dental Institute, King's College London, London, United Kingdom; Duke University Medical Center, United States of America

*Candida* species are one of the most common fungal pathogens of humans and the causative agents of superficial and invasive candidiasis. The vast majority of *Candida* infections are mucosal, manifesting as vaginal or oral candidiasis, which together account for an estimated 40 million infections per year. High-level *Candida* colonisation is also associated with several gut diseases, including Crohn's disease and ulcerative colitis, and reducing fungal burdens reduces disease severity [1]. Additionally, *Candida* species are an ever-increasing problem in immunocompromised patients. Furthermore, in common with the vast majority of life-threatening systemic infections, systemic *Candida* infections are usually acquired through mucosal surfaces. Therefore, it is of paramount importance to understand how epithelial tissues detect and restrict these pathogens to mucosal surfaces.

## Which *Candida albicans* Factors Are Required for Mucosal Infections?

The most common *Candida* species that causes human mucosal infections is *Candida albicans*, an endogenous commensal in approximately 50% of individuals. *C. albicans* is able to undergo morphological switching between a yeast and hyphal form, and the ability to switch to the hyphal form is a critical feature of pathogenicity at mucosal surfaces. Several unique hyphal proteins such as hyphal wall protein 1 (Hwp1p) and agglutinin-like sequence 3 (Als3p) have been identified as virulence attributes by promoting epithelial attachment and invasion [2]. In addition, other virulence factors that promote *C. albicans* pathogenicity (e.g., biofilm formation, hydrolytic enzyme production) are also linked to hypha formation. Furthermore, *C. albicans* strains unable to produce hyphae or maintain hypha formation are non-invasive and avirulent in vitro and in murine models of mucosal infection [3], [4], indicating a key role for hypha formation in *C. albicans* pathogenicity.

## How Is *C. albicans* Recognised by Epithelial Cells?

Mucosal surfaces comprise epithelial cells, which are the first line of defence against *C. albicans*. However, the epithelial receptors that trigger immune responses in response to this fungus are largely unknown. In oral epithelial cells, recognition of yeast and hyphal cells can occur via conventional fungal pathogen-associated molecular patterns (PAMPs) (e.g., mannans, β-glucans) and pattern recognition receptors (PRRs) (e.g., toll-like receptors, C-type lectin receptors), but activation of an immune response appears to be independent of these PAMPs and PRRs [5]. Rather, *C. albicans* appears to interact with epithelial-associated proteins such as E-cadherin and human epidermal growth factor receptor 2 (Her2) [6]. This recognition event triggers the induced endocytosis of *C. albicans*, providing a mechanism of epithelial cell entry and promoting pathogenicity. Although endocytosis is required for invasion, there is only circumstantial evidence to suggest that endocytosis directly contributes to immune activation [7]. Accordingly, the epithelial PRRs or receptors involved in the induction of pro-inflammatory responses by *C. albicans* remain to be elucidated.

## How Is “Pathogenic” *C. albicans* Identified?

**Figure 1:**
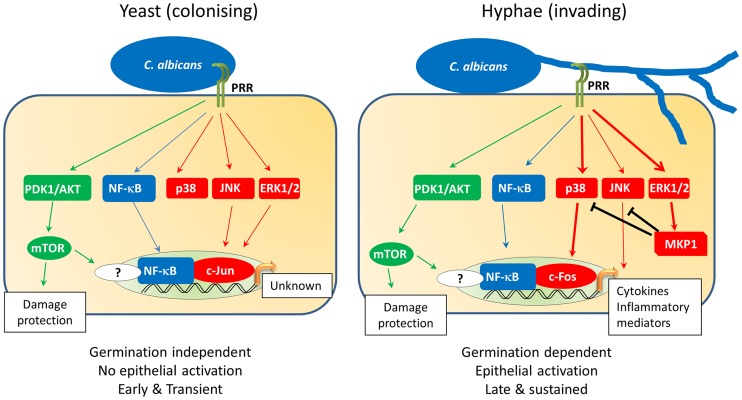
*C. albicans*-induced epithelial cell signalling. Oral epithelial cell discrimination of *C. albicans* yeast from hyphae is enabled via differential MAPK signalling. Recognition of yeast triggers activation of PI3K (green) and NF-κB (blue) as well as weak, transient activation of all three MAPK pathways (red). This MAPK activation leads to a transient activation of the c-Jun transcription factor via JNK/ERK1/2 signalling, with as-yet-unknown transcriptional effects. Activation of the PI3K pathway leads to activation of the epithelial damage protection and/or prevention response. Exposure of epithelial cells to *C. albicans* hyphae leads to the strong activation of MAPK signalling, resulting in the activation of the c-Fos transcription factor via the p38 pathway. At this point, regulation of MAPK signalling is initiated by the induction and stabilisation of the MAPK phosphatase, MKP1 (via the ERK1/2 pathway), which acts to regulate p38 and JNK signalling. Activation of c-Fos in the presence of NF-κB and PI3K signalling leads to the production of cytokines and inflammatory mediators, thereby activating immune responses to *C. albicans*.

Given that mucosal surfaces are in constant contact with the microbiome, an important function of epithelial cells is to respond to opportunistic microbes when they become pathogenic in order to raise an appropriate host response. This is achieved predominantly through the activation of cellular signalling mechanisms, including mitogen-activated protein kinase (MAPK), nuclear factor kappa-light-chain-enhancer of activated B cells (NF-κB) and phosphatidylinositide 3-kinase (PI3K) pathways. With regard to *C. albicans*, oral and vaginal epithelial cells detect both the yeast and hyphal form of this fungus (Figure 1) [5], [8], [9]. Despite not inducing damage, *C. albicans* yeast cells weakly activate all three MAPK pathways (p38, c-Jun N-terminal kinases [JNK], extracellular signal-regulated protein kinases 1 and 2 [ERK1/2]), together with NF-κB and PI3K signalling. This drives the activation of the transcription factors NF-κB and c-Jun (via ERK1/2 and JNK) but is insufficient to induce immune activation (Figure 1). In contrast to yeast cells, *C. albicans* hyphae also activate MAPK signalling but specifically induce the transcription factor c-Fos (via p38). Furthermore, *C. albicans* hyphae activate MAPK phosphatase 1 (MKP1) via the ERK1/2 pathway, which is known to regulate MAPK-mediated immune responses. This combination of c-Fos and MKP1 activation is specifically associated with hypha formation and correlates with immune activation. In contrast, activation of the PI3K pathway is involved in protection against hypha-induced damage. While hypha-induced damage triggers host immune defences, the mechanisms that induce these protective PI3K signals are currently unknown.

## How Does Epithelial Activation Induce Innate Immunity?

**Figure 2:**
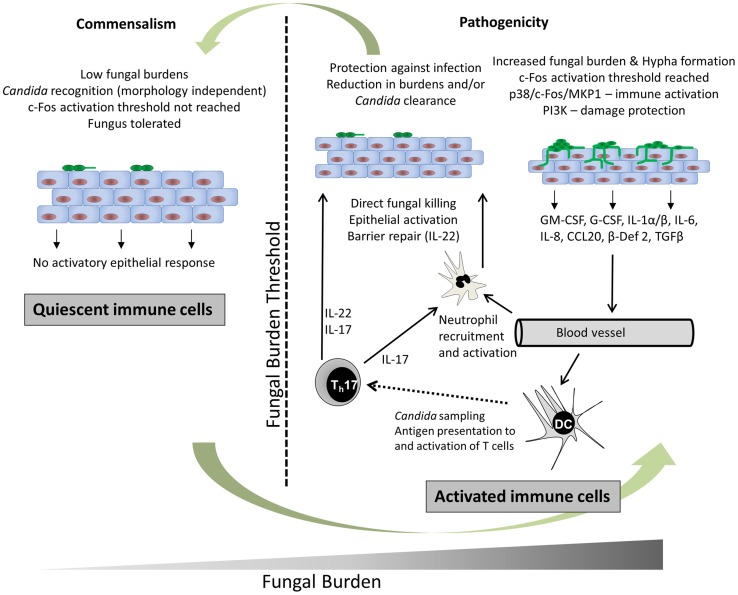
*C. albicans* recognition and protection at mucosal surfaces. In health (left panel) *C. albicans* resides in the commensal state, which is characterised by low fungal burdens. *C. albicans* is recognised but an activation threshold is not reached; thus, the fungus is tolerated without activating epithelial immune responses. During infection (right panel), *C. albicans* burdens increase and an activation threshold is reached when a sufficient hyphal biomass is present. Immune recognition of *C. albicans* hyphae occurs via unknown PRR mechanisms but results in the activation of NF-κB, MAPK, and PI3K signalling pathways. Signalling via p38/c-Fos enables discrimination between yeast and hyphae whilst all three pathways (NF-κB, MAPK, and PI3K) promote immune activation, particularly via p38/c-Fos. Finally, PI3K signalling activates epithelial damage protection/prevention mechanisms. Cytokines and chemokines secreted by epithelial cells in response to *C. albicans* hypha invasion and damage recruit and activate immune cells. IL-8 recruits neutrophils that are in turn activated by GM-CSF, G-CSF, and IL-1 family members. Neutrophils protect directly through phagocytosis and NET (neutrophil extracellular trap) formation and indirectly via immunological cross-talk with epithelial TLR4. CCL20 and β-defensin 2 secretion recruits mucosal-homing CCR6-expressing dendritic cells, which will process fungal antigens and activate Th immunity, including Th17 cells. TGFβ may also act with IL-1α and IL-6 to induce Th17 differentiation. IL-17 production by Th17 cells increases neutrophil activity and IL-22 production promotes epithelial barrier function. Together, these innate and adaptive immune response mechanisms ultimately clear the fungus or reduce fungal burdens below the activation threshold, thereby re-establishing the commensal phenotype.

Epithelial activation by *C. albicans* hyphae induces pro-inflammatory immune responses, which results in the recruitment of innate immune cells, particularly neutrophils (Figure 2). Neutrophils protect against *C. albicans* infection directly through phagocytosis and neutrophil extracellular trap (NET) formation [10] and indirectly via immunological cross-talk with the epithelium [11]. However, neutrophils do not play an obvious protective role during vaginal infection and might even exacerbate disease in humans [12]. Other key epithelial responses include the production of antimicrobial peptides (e.g., β-defensins, cathelicidin), alarmins (e.g., S100A8/9), and matrix metalloproteases; together these help combat fungal infections, initiate other immune responses, and promote epithelial remodelling and barrier repair [13]. In addition, oral and vaginal epithelial cells possess direct antifungal activity [14], suggesting that the uppermost epithelial layers are able to naturally prevent *C. albicans* hypha formation and growth at mucosal surfaces, thereby helping to maintain *C. albicans* in the commensal state.

## How Does Epithelial Activation Induce Adaptive Immunity?

Epithelial cells initiate adaptive immunity via the production of pro-inflammatory molecules, which act as chemoattractants to recruit mucosal-homing dendritic cells. Dendritic cells recognise *C. albicans* through PAMPs such as mannans and β-glucan using conventional PRRs, which ultimately results in the activation of different T helper cells (e.g., Th1, Th2, Th17, Tregulatory) in the local draining lymph nodes (Figure 2). The most recent advances in adaptive fungal immunity relate to the recently identified Th17 cells and many of the functions previously ascribed to Th1 cells are now regarded as being functions of this new T cell phenotype. Th17 cells secrete interleukins IL-17A and IL-17F, which stimulate a variety of cells (e.g., epithelial cells and fibroblasts) to produce antimicrobial peptides, metalloproteases, and chemokines, which promotes neutrophil recruitment and activation [15], ultimately resulting in fungal clearance. Th17 cells also secrete IL-22, which limits fungal growth and maintains epithelial barrier function (Figure 2) [16]. Notably, patients with impaired IL-17 production or hyper-IgE syndrome (HIES) are unable to clear mucosal *C. albicans* infections and develop chronic mucocutaneous candidiasis [17]. Indeed, many studies investigating patients with autoimmune conditions have highlighted the importance of Th17 responses in protection against mucosal candidiasis.

## Summary

Mucosal immune responses to *C. albicans* are highly diverse because of the variety of fungal PAMPs and antigens recognised by different host cells at multiple infection sites. The key function of epithelial cells appears to be discrimination between the morphological status and between the potentially commensal and pathogenic states of *C. albicans*. Epithelial activation initiates a complex network of immune interactions between host and fungus, which determines the downstream innate and adaptive response that ultimately resolves the infection. Whilst much progress has been made in deciphering the key proteins, cells, and mechanisms contributing to host immunity against *Candida*, the next few years should provide a leap forward in clinical and translational applications with regard to how *Candida* infections can be managed and controlled at mucosal surfaces without recourse to antimycotic agents.
